# Endoscopic versus percutaneous drainage of symptomatic pancreatic fluid collections: a 14-year experience from a tertiary hepatobiliary centre

**DOI:** 10.1007/s00464-015-4668-x

**Published:** 2015-12-16

**Authors:** Margaret G. Keane, Shun Fung Sze, Natascha Cieplik, Sam Murray, Gavin J. Johnson, George J. Webster, Douglas Thorburn, Stephen P. Pereira

**Affiliations:** 1 Institute for Liver and Digestive Health, Royal Free Hospital, University College London, Pond St, London, NW3 2PF UK; 2 Department of Gastroenterology, University College London Hospitals NHS Foundation Trust, 235 Euston Road, London, NW1 2BG UK

**Keywords:** Acute pancreatitis, Chronic pancreatitis, Pseudocyst, Walled-off pancreatic necrosis, Endoscopic ultrasound, Endoscopic drainage

## Abstract

**Introduction:**

Endoscopic transmural drainage (ED) or percutaneous drainage (PD) has mostly replaced surgery for the initial management of patients with symptomatic pancreatic fluid collections (PFCs). This study aimed to compare outcomes for patients undergoing ED or PD of symptomatic PFCs.

**Methods:**

Between January 2000 and December 2013, all patients who required PD or ED of a PFC were included. Rates of treatment success, length of hospital stay, adverse events, re-interventions and length of follow-up were recorded retrospectively in all cases.

**Results:**

In total, 164 patients were included in the study; 109 patients underwent ED; and 55 had PD alone. During the 14-year study period, the incidence of ED increased and PD fell. In the 109 patients who were managed by ED, treatment success was considerably higher than in those managed by PD (70 vs. 31 %). Rates of procedural adverse events were higher in the ED cohort compared to the PD group (10 vs. 1 %), but patients managed by ED required fewer interventions (median of 1.8 vs. 3.3) had lower rates of residual collections (21 vs. 67 %) and need for surgical intervention (4 vs. 11 %). In the ED group, treatment success was similar for walled-off pancreatic necrosis (WOPN) and pseudocysts (67 vs. 72 %, *P* = 0.77). There were no procedure-related deaths.

**Conclusion:**

Compared with PD, ED of symptomatic PFCs was associated with higher rates of treatment success, lower rates of re-intervention, including surgery and shorter lengths of hospital stay. Outcomes in WOPN were comparable to those in patients with pseudocysts.

Pancreatic fluid collections (PFCs) are collections of pancreatic fluid or debris that are encased in a wall of granulation tissue. They occur following acute pancreatitis, pancreatic surgery, abdominal trauma or chronic obstruction of the pancreatic duct, e.g. in chronic pancreatitis or pancreatic malignancy [1–3]. PFCs are estimated to occur in 5–16 % [4] of patients with acute pancreatitis and up to 40 % of patients with chronic pancreatitis [5].

While most asymptomatic inflammatory pancreatic collections, especially if small (e.g. <4 cm), will resolve spontaneously and can be managed conservatively [6], once a PFC increases to 6 cm or becomes symptomatic (e.g. infection, abdominal pain, biliary or gastric outlet obstruction), rates of spontaneous resolution are much lower and drainage is recommended [7–11]. The management of asymptomatic PFCs larger than 6 cm remains debated, but conservative management is often advocated given the potential for procedure-associated morbidity in an asymptomatic patient [10].

PFCs may be drained surgically, by radiologically guided percutaneous drainage (PD), or endoscopically, usually by endoscopic transmural drainage (ED). Historically, surgery was considered the standard initial management for PFCs, but given its invasive nature, morbidity, longer length of hospital stay and increased associated costs, there has been a growing interest in less invasive approaches for the management of PFCs, such as PD or ED [2, 3, 12–15].

The first reports of the endoscopic creation of a fistulous tract between a PFC and the gastrointestinal tract were published in the 1980s [16–18]. Since these early descriptions, the technique has evolved and now routinely combines endoscopic ultrasound (EUS) enabling the drainage of non-bulging collections and reducing associated adverse events [2, 3, 19–23.]

Previous studies have compared surgical drainage with PD [14, 24–27] and ED [13]. One group compared all three approaches, but in this study, only a few patients underwent PD, so the final analysis focused on outcomes for surgical and ED [28]. A subsequent study did compare outcomes for PD and ED, but focused on outcomes in pseudocysts [29]. Increasingly, necrotic PFC are being managed by minimally invasive approaches given the morbidity associated with open surgical interventions [2, 3, 30], but there remains a lack of information about the optimal minimally invasive drainage method in different PFCs.

## Study aim

The aim of this study is to compare the outcomes of endoscopic or percutaneous drainage in patients with symptomatic PFC.

## Methods

### Setting

A large regional hepatopancreaticobiliary centre based across two tertiary care hospitals: University College London Hospital NHS Foundation Trust (UCLH) and the Royal Free Hospital NHS Foundation Trust (RFH).

### Design

This is a retrospective cohort study.

### Ethical approval

The study was registered as a clinical audit at University College London NHS Foundation Trust and the Royal Free Hospital NHS Foundations Trust and conducted in accordance with the Helsinki Declaration [31].

### Definitions

#### Pancreatic fluid collection (PFC)

PFC in this study was defined as measuring >4 cm on CT/MRCP and located within or adjacent to the pancreas, in patients with a documented history of acute or chronic pancreatitis, pancreatic surgery or malignancy.

#### Types of PFCs

PFCs were defined in accordance with the revised Atlanta criteria: [1]

- **Pseudocyst** collection of fluid encapsulated within a well-defined inflammatory wall containing no solid components or necrosis. Present for >4 weeks.
- **Walled-off pancreatic necrosis (WOPN)** mature, encapsulated collection of pancreatic ± peripancreatic necrosis that has a well-defined inflammatory wall. Present for >4 weeks.

#### Treatment success

Treatment success was defined as the successful insertion of a stent or drain with complete resolution or a decrease in the size of the PFC to ≤2 cm on follow-up CT.

#### Residual PFC

A residual collection was defined as the presence of a PFC (>2 cm), which did not resolve on imaging following percutaneous or endoscopic drainage.

#### Recurrent PFC

Recurrence was defined as the presence of a PFC on imaging after resolution of the initial collection.

#### Re-intervention

Re-intervention was defined as the need for repeat drainage or surgery owing to persistent symptoms in association with a residual PFC on follow-up imaging.

#### Length of stay

Length of hospital stay was defined as the time to discharge from the day of the first percutaneous drainage (percutaneous management group) or first endoscopic transmural drainage (endoscopic management group).

#### Infected collection

An infected PFC was diagnosed based on fever, raised inflammatory markers, radiological findings (e.g. gas bubbles within the PFC) or positive culture from the PFC following aspiration.

### Procedure: EUS-guided transmural drainage

Prior to EUS, cross-sectional imaging (computed tomography (CT) or magnetic resonance imaging (MRI)/magnetic resonance cholangiopancreatography (MRCP)) was performed within 4 weeks, to determine the size and location of the cyst, as well as to evaluate for interposed vascular structures or evidence of pancreatic duct disruption necessitating a pancreatic stent.

The procedures were performed under conscious sedation or general anaesthesia, a linear array echoendoscope (Pentax Medical, UK or Olympus, UK) was used to ensure the distance between the gastric and/or duodenal wall, the PFC was <1 cm, and there were no interposed blood vessels on Doppler.

In the majority of cases, the PFC was accessed from the stomach using a cystotome (Cook Medical, Limerick, Ireland). In other cases, access was obtained using a needle knife or 19-gauge fine needle aspiration needle (ECHO-19; Cook Medical or Expect; Boston Scientific, Hemel Hempstead, UK). Entry was confirmed by aspiration of cyst contents, after which two 0.035-inch guidewires were then advanced into the PFC and allowed to coil within the cyst under fluoroscopic guidance, which was used in all cases. The tract was then dilated with a controlled radial expansion (CRE) wire-guided balloon (Boston Scientific) or Soehendra biliary dilator. Usually, two double-pigtail stents (7F) of various lengths were then inserted into the fistulotomy using a Teflon pusher catheter (Cook Medical). Cyst fluid was obtained and sent for Gram stain, culture and fluid amylase levels as clinically indicated.

Patients were discharged when clinically stable (aim within 24 h) and prescribed a short course of oral antibiotics for up to 5 days. They were then followed up in clinic 3–6 monthly as necessary. Transmural stents were generally removed 9–12 months after insertion, as long as the PFC had resolved on cross-sectional imaging. If patients remained symptomatic, and the PFC persisted or recurred, additional drainage was performed following discussion at the hepatopancreaticobiliary (HPB) multidisciplinary meeting.

### Procedure: percutaneous drainage

Percutaneous drainage was performed by a HPB radiologist under CT, ultrasound (US) and/or fluoroscopic guidance. The PFC was identified, and a suitable access route selected, which avoided the spleen, interposed bowel and blood vessels. The skin was marked and local anaesthesia administered (subcutaneous injection of 1 % lidocaine). The PFC was then punctured under imaging guidance with an 18-gauge single-wall needle (Cook Medical). A Seldinger technique was then used to sequentially dilate the tract over a 0.035-inch, non-hydrophilic guidewire (Cook Medical). A multiple-side holed 8F–12F (Flexima APDL; Boston Scientific) locking catheter was then placed and secured to the skin with 2-0 nylon suture. Drain output was monitored daily, and decisions about upsizing, replacing or removing the drain were made based on subsequent imaging and the clinical progress of the patient.

### Inclusion criteria

Patients with a PFC requiring percutaneous or endoscopic transmural drainage between 1 January 2000 and 31 December 2013.

### Exclusion criteria

- Patients <18 years.
- PFC <4 cm in size or managed conservatively.
- Patients who were managed by surgery (*n* = 13), EUS-guided fine needle aspiration (EUS-FNA) (*n* = 7) or endoscopic retrograde cholangiopancreatography (ERCP) and transpapillary drainage (*n* = 2) alone.

### Data recorded

Cases were identified primarily from records of the benign HPB multidisciplinary team meetings, which are held weekly. In addition, the Pathology (CoPath histology database, Sunquest, Tucson AZ, USA), Endoscopy (GI reporting tool, Unisoft medical systems, UK) and Imaging (PACS: picture archiving and communication system, GE Healthcare, USA) databases were searched using the following terms, pseudocyst, walled-off necrosis, pancreatic fluid collection.

The electronic medical records of the included patients were reviewed, and information was recorded in an electronic spreadsheet. Data collected included demographic information (age, sex, hospital number), initial symptoms, history of acute or chronic pancreatitis or malignancy, family history of pancreatic cancer or relevant clinical syndrome and serum amylase on admission, where available. Cross-sectional imaging (CT and/or MR/MRCP) features that were recorded included size (maximal dimension), location, number of cystic lesions, presence of a necrosis, features of acute or chronic pancreatitis, dilatation of the pancreatic duct or biliary tree, communication of the cystic lesion with the main pancreatic duct, ascites, pleural effusion or the presence of features of portal hypertension. For patients undergoing endoscopy (ERCP or EUS), imaging features were recorded in addition to details of the drainage technique and cytology, histology or culture results where available. For patients ultimately referred for surgery, date of the operation, type of resection and final histology were recorded. Length of follow-up was calculated from first procedure to last clinic appointment attended, or date of clinic discharge, or death.

### Statistical analyses

Statistical Package for Social Sciences for Windows, version 18.0 (SPSS Inc., Chicago, IL, USA), was used to perform all statistical analyses. Associations between malignancy and various clinical and radiographic characteristics were evaluated using a two-sample *t* test for continuous variables and a Chi-squared test for categorical variables.

## Results

**Fig. 1:**
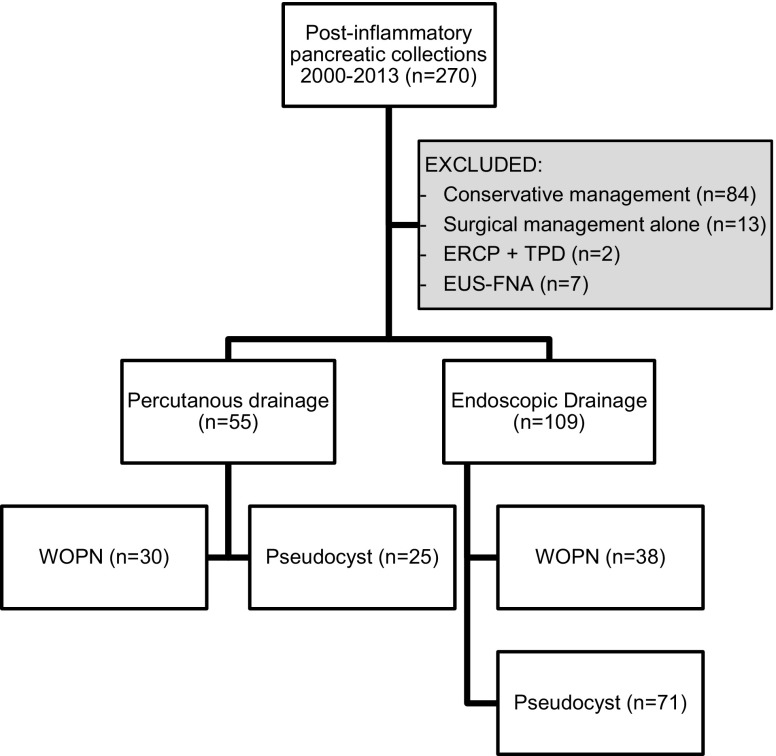
Flow chart demonstrating patient selection and proportion of patients with pseudocysts and WOPN in each cohort. *WOPN* walled-off pancreatic necrosis*, ERCP* + *TPD* endoscopic retrograde cholangiopancreatography + transpapillary drainage, *EUS*-*FNA* endoscopic ultrasound and fine needle aspiration

Between January 2000 and December 2013, 270 patients with a PFC were evaluated at UCLH and the RFH. In total, 84 patients were managed conservatively and 13 by surgery alone and were excluded from the study (Fig. 1). Two patients had ERCP and transpapillary drainage and seven EUS-guided fine needle aspiration (EUS-FNA) to dryness alone; these cases were also excluded. The 7 patients having EUS-FNA alone were initially referred for consideration of ED, but at the time of the procedure, ED was not performed because of small cyst size (*n* = 2) or phlegmon containing largely solid material (*n* = 5).

**Fig. 2:**
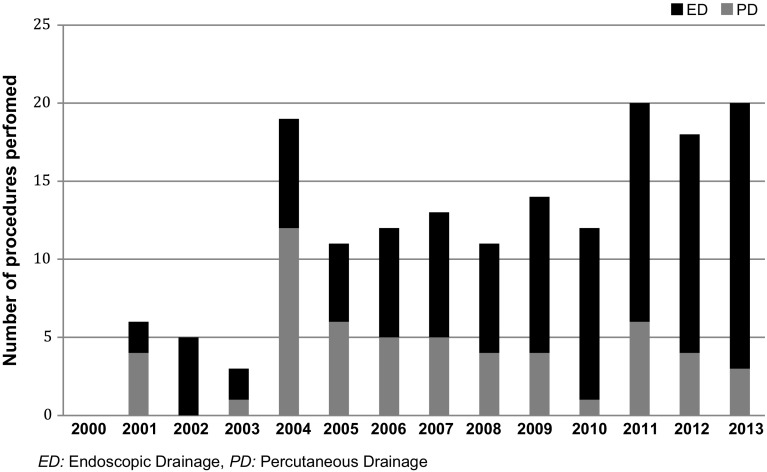
Number of patients undergoing drainage of a pancreatic fluid collection annually during the study period

Of the 164 patients included in the study, 109 patients underwent ED and 55 patients had PD alone (Fig. 1). During the study period, the overall number of patients undergoing drainage of their PFC increased annually, and the number of patients managed by PD alone decreased in favour of ED for both first-line and definitive management of a PFC (Fig. 2).

### Diagnostic investigations

#### Clinical features and laboratory tests

Patient demographics are outlined in Table 1. The median age of patients undergoing ED was 55 years (range 22–84 years) compared to 50 years (range 20–87 years) for those receiving PD alone. Both groups had more male than female patients; 55 % (60) in the ED group and 67 % [37] in the PD group. The most common aetiologies of pancreatitis in both groups were gallstones and alcohol. In the ED group, more patients had gallstone pancreatitis (45 vs. 26 %; *P* = 0.01), and in the PD group, slightly more patients had alcohol-related pancreatitis (24 vs. 19 %). The cause of pancreatitis remained indeterminate in 25 % of the ED group and 38 % of those who underwent PD alone.

**Table 1:** Characteristics of patients undergoing drainage of PFCs by management subtype

	Endoscopic transmural drainage (*n* = 109)	Percutaneous drainage (*n* = 55)
Median age of patient, years (range)	55 (22–84)	50 (20–87)
Sex		
Male	55 % (60)	67 % (37)
Female	45 % (49)	33 % (18)
Aetiology		
Gallstones	45 % (50)	26 % (14)
Alcohol	19 % (21)	24 % (13)
Post-ERCP or EUS	3 % (3)	6 % (3)
Hypercalcaemia	1 % (1)	2 % (1)
Alcohol + gallstones	2 % (2)	–
Hyperlipidaemia	1 % (1)	–
Pancreatic/ampullary tumour	1 % (1)	2 % (1)
Post-pancreatic surgery	1 % (1)	2 % (1)
Tuberculosis	–	2 % (1)
Incidental finding	2 % (2)	–
Indeterminate	25 % (27)	38 % (21)

#### Cross-sectional imaging

Abdominal CT was used to confirm the presence of a PFC in all patients. Both CT and MRCP/MRI were performed in 46 % (76/164) of patients. The median size of the PFC managed by ED or PD was similar; 103 versus 102 mm, respectively. In both groups, approximately two-thirds of the collections were located within or adjacent to the body or tail of the pancreas. In total, 32 % of those undergoing ED and 42 % of those receiving PD had evidence of chronic pancreatitis on cross-sectional imaging immediately prior to drainage. The ED group contained fewer patients with necrotising pancreatitis (35 vs. 55 %). Evidence of portal hypertension on imaging was common in both groups, 49 % in the ED group and 42 % in the PD group (Table 2).

**Table 2:** Comparison of cross-sectional imaging features by management subtype

	Endoscopic transmural drainage (*n* = 109)	Percutaneous drainage (*n* = 55)
Median size of PFC + range (mm)	103 (40–250)	102 (40–222)
Site		
Head/neck	37 % (41)	36 % (20)
Body/tail	63 % (68)	64 % (35)
Acute pancreatitis	73 % (80)	76 % (42)
Chronic pancreatitis	32 % (35)	42 % (23)
Pancreatic necrosis	35 % (38)	55 % (30)
Pancreatic duct dilatation	39 % (43)	40 % (22)
Extrahepatic biliary dilation	35 % (38)	38 % (21)
Pseudoaneurysm	6 % [6 (2 haemorrhages)]	11 % [6 (4 haemorrhages)]
Ascites	11 % (12)	31 % (17)
Pleural effusion	16 % (17)	40 % (22)
Portal hypertension	49 % (53)	42 % (23)
Multiple pancreatic cysts	23 % (25)	35 % (19)
Lymph node enlargement	19 % (21)	13 % (7)

#### Management and outcomes

Almost all drains were placed more than 4 weeks after the onset of pancreatitis, with the exception of a few patients with an infected PFC. In the PD group, the median time to first drainage was 28 days (range 1–1444 days) compared to 101 days (range 7–3183 days) in the ED group.

Of the ED procedures undertaken, 35 % were performed in patients with WOPN (Table 3). Patients with WOPN undergoing ED were older than the pseudocyst group (60 vs. 51 years), had larger collections (119 vs. 100 mm) and had higher rates of portal hypertension (58 vs. 44 %). All patients had symptoms attributable to their cyst, and the most common indication for drainage was pain. Clinical signs of infection were more frequent in the WOPN group (76 vs. 37 %). When fluid was aspirated from a PFC for culture, it was almost always positive (WOPN 96 vs. pseudocysts 100 %). Most fluid aspirates had mixed growth (WOPN 46 vs. pseudocysts 50 %), and patients with WOPN were co-infected with methicillin-resistant Staphylococcus aureus (MRSA) and candida in 17 and 25 % of cases, respectively, compared to 5 and 9 % in pseudocysts (Table 3).

**Table 3:** Comparison of clinical outcomes of endoscopic transmural drainage in walled-off pancreatic necrosis (WOPN) and pseudocysts

	WOPN Patients = 38 Procedures = 46	Pseudocyst Patients = 71 Procedures = 81
Median age of patient, years (range)	60 (22–84)	51 (26–84)
Median maximal diameter—mm (range)	119 (67–200)	100 (40–250)
Portal hypertension % (*n*)	58 % (22)	44 % (31)
Reason for drainage % (*n*)		
Pain	24 % (11)	43 % (35)
Increasing size + pain	35 % (16)	32 % (26)
Infection	28 % (13)	11 % (9)
Gastric outlet obstruction	7 % (3)	6 % (5)
Unknown	7 % (3)	7 % (6)
Sedation % (*n*)		
Conscious sedation	97 % (37)	98 % (58)
General anaesthesia	3 % (1)	2 % (1)
Unknown	(8)	(23)
Approach % (*n*)		
Transgastric	98 % (42)	91 % (60)
Transduodenal	2 % (1)	9 % (6)
Unknown	(3)	(15)
Cystotome used 5 (*n*)	74 % (34)	67 % (54)
Number of stents inserted % (*n*)		
Plastic		
0 (Failed stent insertion/procedure abandoned)	2 % (1)	10 % (8)
1	9 % (4)	10 % (8)
2	70 % (32)	62 % (50)
3	4 % (2)	6 % (5)
4	2 % (1)	0 % (0)
Unknown	7 % (3)	10 % (8)
Fully covered self-expanding metal stents (FCSEMS)	7 % (3)	3 % (2)
Infection % (*n*)		
Clinical signs of infection	76 % (29/38)	37 % (26/71)
Positive pancreatic fluid culture	96 % (23/24)	100 % (22/22)
Mixed growth	46 % (11)	50 % (11)
Streptococcus	0 % (0)	18 % (4)
Staphylococcus	17 % (4)	14 % (3)
*E coli*	13 % (3)	5 % (1)
Enterococcus	8 % (2)	5 % (1)
Other	16 % (4)	10 % (2)
Pancreatic fluid—co-infection		
MRSA	17 % (4)	5 % (1)
Candida	25 % (6)	9 % (2)
Treatment success % (*n*)	67 % (31/46)	72 % (58/81)
Failed stent insertion/procedure abandoned	2 % (1/46)	10 % (8/81—2 cases required a further procedure)
Further procedure required	37 % (14/38)	24 % (17/71)
Adverse events % (*n*)	7 % (3)	12 % (10)
Stent migration	2 % (1)	4 % (3)
Haemorrhage—transfusion required	0 % (0)	3 % (2:1—acute, 1—delayed)
Pneumoperitoneum	2 % (1)	3 % (2)
Oesophageal perforation	0 % (0)	3 % (2)
Aspiration pneumonia	2 % (1)	0 % (0)
Pneumothorax	0 % (0)	1 % (1)
Residual PFC % (*n*)	22 % (10/46)	20 % (16/81)
Recurrent PFC % (*n*)	9 % (4/46)	5 % (4/81)
Number of drainage interventions required		
Mean number of interventions (range)	2.3 (1–7)	1.5 (1–4)
Mean pre-ED (range)	0.9 (0–6)	0.3 (0–3)
Mean post-ED (range)	0.5 (0–3)	0.3 (0–3)
Surgical intervention ultimately required—% (*n*)	5 % (2)	3 % (2)
Length of stay: median number of days in hospital post-ED (range)	4 (0–36)	4 (0–63)

Almost all (98 % (94/96)) ED were performed under conscious sedation. The puncture was attempted through the transgastric route in 98 % of the WOPN and 91 % of pseudocysts. A 10F cystotome (Cook Medical) was used in approximately two-thirds of cases. Stent insertion was successful in 98 % of WOPN and 90 % of pseudocysts. A median of two plastic double-pigtail stents (range 1–4) were inserted. A fully covered self-expanding metal stent (FCSEMS) (NAGI stent, Taewoong Medical, Gyeonggi-do, Korea) was inserted in 4.6 % (5/109) of cases. Treatment success was similar in both the WOPN and pseudocyst groups (67 % (31/46) vs. 72 % (58/81), *P* = 0.77). The median length of stay post-ED was 4 days and did not differ between patients with pseudocysts and WOPN collections, nor did the need for a further procedure (31 vs. 21 %, *P* = 0.11). Adverse events occurred in 10 % of cases and were similar in both the WOPN and pseudocyst groups. Adverse events included four episodes of stent migration, three cases of pneumoperitoneum (one managed conservatively with percutaneous drainage, while two underwent laparotomy), two oesophageal perforations which required laparotomy, two episodes of gastrointestinal bleeding requiring blood transfusion and endoscopic or radiological intervention, one pneumothorax which required drainage and one aspiration pneumonia. No patients died in the 30 days following the procedure.

In the 55 patients who were managed by PD, 18 % (10/55) of the patients had the drain placed transgastrically, of which 8 were internalised. Treatment success in the PD group was considerably lower than those managed by ED [31 % (30/97) vs. 70 % (89/127)]. During the median follow-up period of 11 (0–131) months in the ED group and 17 (1–150) months in the PD group, rates of failed drain insertion and adverse events were lower in the PD cohort compared to the ED group, 1 and 1 % versus 7 and 10 %, respectively. However, patients managed by PD alone required more interventions (median of 3.3 vs. 1.8), had higher rates of residual collections (67 vs. 21 %) and ultimately a higher proportion required surgical management (11 vs. 4 %) and had a longer hospital stay (median 42 vs. 4 days) (Table 4).

**Table 4:** Comparison of clinical outcomes following endoscopic transmural or percutaneous drainage

	Endoscopic transmural drainage Patients = 109 Procedures = 127	Percutaneous drainage Patients = 55 Procedures = 97
Treatment success % (n)		
Overall	70 % (89/127)	31 % (30/97)
WOPN	67 % (31/46)	23 % (15/65)
Pseudocyst	72 % (58/81)	47 % (15/32)
Failed drain/stent insertion % (*n*):		
Overall	7 % (9)	1 % (1)
WOPN	2 % (1)	2 % (1)
Pseudocyst	10 % (8)	0 % (0)
Rate of adverse events % (n)	10 % (13)	2 % (2)
Adverse events	Stent migration (4) Pneumoperitoneum (3)—*percutaneous drainage* *×* *1 and a laparotomy* x2 Oesophageal perforation (2)—*both required laparotomy* Gastrointestinal bleeding (2)—*both required transfusion and endoscopic or radiological intervention* Pneumothorax (1)—*chest drain* Aspiration pneumonia (1)	Pancreatic fistula (2)—both managed conservatively
Median hospital stay post-procedure (range)	4 (0–63)	42 (2–199)
30-day mortality % (n):	0 % (0)	7 % (4)—all due to the complications of severe acute pancreatitis
Residual PFC % (*n*)		
Overall	21 % (26)	67 % (65)
WOPN	22 % (10)	74 % (48)
Pseudocyst	20 % (16)	53 % (17)
Recurrent PFC % (*n*):	6 % (8)	2 % (2)
Number of drainage interventions required:		
Mean number of interventions (range)	1.8 (1–7)	3.3 (1–11)
Mean pre-ED (range)	0.5 (0–6)	1.1 (0–10)
Mean post-ED (range)	0.3 (0–3)	1.2 (0–10)
Surgical intervention ultimately required	4 % (4)	11 % (6)

Patients managed by percutaneous drainage had an external drain in situ for a median of 56 days (range 3–651 days). Cystenterostomy stents were left in situ for a median of 277 days (range 20–1015 days) before removal.

## Discussion

Historically, PFCs have been managed by surgical drainage. However, the associated morbidity and mortality of pancreatic surgery have resulted in a growing interest in alternative minimally invasive techniques. A recent randomised study of 40 patients compared ED and surgical drainage. ED was found to have comparable efficacy with similar rates of adverse events but with a shorter hospital stay [15]. In the present study, ED was associated with higher rates of treatment success (70 vs. 31 %), a shorter length of hospital stay and fewer subsequent interventions, when compared to PD.

When compared to other case series, the rate of initial technical success was similar, but overall treatment success in this study (70 %) was lower than previously reported [3, 30]. However, it was comparable to series from centres with a longer follow-up time allowing sufficient time to diagnose recurrent or residual collections [32] and those series which included a high proportion of complex cases (e.g. WOPN and portal hypertension) [2, 3]. Other groups have reported much lower rates of treatment success when managing necrotic collections (25 vs. 93 %) [2], but in this study, treatment success rates between WOPN and pseudocysts were comparable (67 vs 72 %). This may reflect differences in technique such as the insertion of more than two plastic stents or the use of FCSEMS in the management of these collections.

Adverse events occurred in 10 % (*n* = 13) of the ED cases, but all were successfully managed during the same hospitalisation. Rates were comparable to those reported in other ED studies and were similar or lower than those following surgical drainage of a PFC [2, 3, 15, 23, 33, 34]. Other authors have also reported higher adverse events when draining organised necrosis [2, 3]. This was not the case in our series, where in fact adverse events were slightly lower in the WOPN cohort compared to the pseudocyst group (7 vs 12 %). The 30-day mortality following ED in this cohort was zero, compared to 0–7 % in other series [2, 3, 15, 20, 23, 35–38].

Bacterial or fungal colonisation and systemic infection are common sequelae of chronic PFC, particularly in the presence of necrosis. As in this series, typically a range of bacteria is isolated and fungal contamination is present in up to a quarter of cases with WOPN [39]. EUS-guided aspiration is therefore increasingly recommended to guide antibiotic management [40]. Although not performed routinely in most centres, it appears to be a useful strategy for guiding antibiotic and antifungal choice in the management of infected PFCs.

In patients in this series managed by PD alone, rates of treatment success were low and further procedures were commonly required, similar to outcomes reported in other PD series [14, 41–45]. PD also requires patients to have a long-term external drain (median time in situ of 56 days), which is commonly disliked by patients and has been associated with the development of chronic pancreatic cutaneous fistulas [14, 41, 42, 44]. In our series, although patients managed by PD had a longer length of stay in hospital, rates of pancreatic cutaneous fistulas were much lower (2 %) than previously reported, which may be because a proportion (18 %) of the percutaneous drains in our series were sited transgastrically, of which most (80 %) were ultimately converted to an endoscopic cystenterostomy.

One of the limitations of our study was that it was conducted retrospectively. Outcomes for the PD group may have been inferior due to the inclusion of patients with more severe disease as evidenced by higher rates of necrosis, ascites, pleural effusions, shorter time to first drainage (28 vs. 101 days), and that four patients died due to the complications of severe pancreatitis in this cohort.

## Conclusions

ED is increasingly employed in the management of PFC. This large series demonstrated superior rates of treatment success, need for subsequent intervention and shorter length of stay in hospital compared to patients managed by PD alone. Outcomes and adverse events were similar for WOPN and pseudocysts, supporting the use of ED in the management of complex PFCs. Further high-quality studies are needed to fully define optimal pathways for the use of ED in the management of PFC.
